# Which Aspects of Postural Control Differentiate between Patients with Parkinson's Disease with and without Freezing of Gait?

**DOI:** 10.1155/2013/971480

**Published:** 2013-06-27

**Authors:** Griet Vervoort, Evelien Nackaerts, Farshid Mohammadi, Elke Heremans, Sabine Verschueren, Alice Nieuwboer, Sarah Vercruysse

**Affiliations:** Department of Rehabilitation Sciences, KU Leuven, Tervuursevest 101/1501, 3001 Leuven, Belgium

## Abstract

This exploratory study aimed to identify which aspects of postural control are able to distinguish between subgroups of patients with Parkinson's disease (PD) and controls. Balance was tested using static and dynamic posturography. Freezers (*n* = 9), nonfreezers (*n* = 10), and controls (*n* = 10) stood on a movable force platform and performed 3 randomly assigned tests: (1) sensory organization test (SOT) to evaluate the effective use of sensory information, (2) motor control test (MCT) to assess automatic postural reactions in response to platform perturbations, and (3) rhythmic weight shift test (RWS) to evaluate the ability to voluntarily move the center of gravity (COG) mediolaterally and anterior-posteriorly (AP). The respective outcome measures were equilibrium and postural strategy scores, response strength and amplitude of weight shift. Patients were in the “on” phase of the medication cycle. In general, freezers performed similarly on SOT and MCT compared to nonfreezers. Freezers showed an intact postural strategy during sensory manipulations and an appropriate response to external perturbations. However, during voluntary weight shifting, freezers showed poorer directional control compared to nonfreezers and controls. This suggests that freezers have adequate automatic postural control and sensory integration abilities in quiet stance, but show specific directional control deficits when weight shifting is voluntary.

## 1. Introduction

Patients with Parkinson's disease (PD) are prone to falling during daily activities. Recurrent falls are a frequent cause of injuries and hospital admissions for patients with PD and an important factor that negatively influences quality of life [1, 2]. The extent of this problem was shown in a meta-analysis of prospective studies that reported that 46% of the patient population with PD had one or more falls in a 3-month time frame [3].

In order to prevent recurrent falls it is important to gain more insight in the underlying deficits. Recently, a number of prediction studies have shown that postural control deficits and freezing of gait (FOG) are powerful determinants of recurrent falls [4, 5]. Although both signs were previously linked to falls, there are, to our knowledge, no conclusive reports on the relationship between postural control deficits and FOG.

FOG is defined as an episodic inability to generate effective stepping while having the intention to walk [6]. It is most commonly experienced during turning and step initiation, but also when faced with spatial constraints, stress, and distraction [6]. A FOG episode can present itself by a significant step size reduction (shuffling gait), knee trembling, or complete akinesia, all leading to a sudden arrest of walking [7]. During freezing, it sometimes happens that the center of gravity (COG) continues to move forward while the feet stop moving. This can lead to imbalance, which cannot be corrected by compensatory steps and therefore increases the risk of falling [1, 7]. This was supported by Jacobs et al. [8], who showed that patients with PD, compared to controls, fail to initiate compensatory stepping and present with FOG-like trembling knee movements when balance was challenged using a sudden forward platform translation. These findings were interpreted as being indicative of a postural control deficit and, more specifically, a failure to couple balance and voluntary locomotor synergies [8].

Postural control deficits in freezers were also reported by Nantel and coworkers [9]. During voluntary weight shifts as part of a repetitive stepping task, freezers showed rapid, small, and inefficient weight transfers between both legs, which were associated with freezing episodes [9]. In addition, both peripheral proprioceptive feedback and central sensory processing abnormalities have been attributed to postural control deficits in PD [10]. Since both FOG and postural control deficits are associated with increased fall risk, elucidating their relationship is an important step in understanding the problem of recurrent falls in PD.

Therefore, the purpose of this exploratory study was to study both sensory and motor aspects of voluntary and automatic postural control using a moveable balance platform in a group of freezers, nonfreezers, and age-matched controls. This enabled objective quantification of sensory organization processes and postural responses to external perturbations (automatic) and voluntary weight shifting to determine if differences exist between freezers and nonfreezers.

We expected freezers to experience more problems in both voluntary and automatic postural control tasks compared to nonfreezers given the reported greater impairment during automatic task performance and underscaled voluntary body weight transfer during repetitive stepping in place [9]. Additionally, we expected both freezers and nonfreezers to perform worse on these tasks compared to controls [11].

## 2. Materials and Methods

### 2.1. Participants

Nineteen patients with PD and 10 age-matched healthy controls participated in this study. All patients were recruited through the University Hospitals Leuven. Patients were included if they had a Hoehn and Yahr (H&Y) stage between II and IV during the “on” state of the medication cycle and were able to stand independently without interfering dyskinesias. Patients with low back pain, orthostatic hypotension, dementia (Mini Mental State Examination < 24), neurosurgical intervention (subthalamic stimulator), and other diseases affecting postural control and/or proprioception were excluded. The patient group consisted of 9 patients with PD experiencing FOG and 10 patients with PD without FOG as confirmed by a score of 1 or higher on the third question of the freezing of gait questionnaire (FOGQ). The freezer and nonfreezer groups were matched for age and disease severity by means of the Hoehn and Yahr (H&Y) stage and Unified Parkinson's Disease Rating Scale (UPDRS) part III. Those patients on levodopa were tested in the “on” state, about 1-2 hours after medication intake. Each participant signed a written informed consent. The research procedures were approved by the local review board according to the declaration of Helsinki.

### 2.2. Experimental Design

The baseline clinical examination consisted of administering the UPDRS (part III, motor subscale) and the FOGQ [12]. Fall frequency in the past three months was determined retrospectively and patients were assigned one of three fall status categories: (1) no falls or near falls, (2) no falls but at least one near fall, and (3) one fall or more in the last 3 months. A fall was defined as an event resulting in a person coming to rest unintentionally on the ground or other level and not as the result of a major intrinsic event or overwhelming hazard [13, 14]. A near fall was defined as any loss of balance without hitting the floor or other lower surface (fall arrested by seeking support) [15].

To account for possible proprioceptive differences between freezers and nonfreezers, position sense was measured with a lower limb matching task. During this task, participants were blindfolded and instructed to match the position of one lower limb with the position of the other limb, which was held in a fixed position by the investigator. Differences in alignment between both limbs (expressed in degrees) were measured for a range of knee angles during 5 trials and expressed as an average [16]. To avoid muscle fatigue, a short period of rest was given between all trials.

Postural control was measured using the SMART EquiTest System (Neurocom International Inc., Clackamas, OR, USA). Three tests were assigned in random order: (1) the Sensory Organization Test (SOT), (2) the Motor Control Test (MCT), and (3) the Rhythmic Weight Shift test (RWS). These tests were selected to address global postural control, that is, static and dynamic postural (automatic and voluntary control), as well as the influence of the various sensory modalities. Participants were allowed to rest between tests to avoid muscle fatigue. They were placed bare-footed on the moveable force plate and were instructed to stand still during SOT and MCT conditions leaving their hands hanging besides their body, looking straight ahead. Foot placement was adapted as a function of body height, so taller participants had a wider base of support. To avoid falls, a harness was fitted onto the participant and a second examiner was standing nearby.

The Sensory Organization Test (SOT) was used to assess postural control and the ability to integrate sensory (visual, vestibular, and proprioceptive) information under 6 systematically manipulated conditions [11]. Each condition consisted of 3 trials of 20 seconds during which the postural sway and the postural strategy (relative amount of ankle to hip movement where 100% indicated ankle movement only and 0% hip movement only) were measured. The outcome measure generated from this test was the equilibrium score, which was averaged for 3 trials of the 6 conditions in which sensory information was manipulated. The equilibrium score is a valid measure for postural stability comparing the participants' sway with their theoretical limits of stability (LOS) (12.5°) calculated by the formula (12.5(*θ*_max⁡_ − *θ*_min⁡_) × 100)/12.5 where *θ* reflects the sway angle in response to the perturbation [17]. A higher equilibrium score represented a better ability to maintain balance.

The Motor Control Test (MCT) assessed the automatic postural reactions in response to platform translations of various sizes (small, medium, and large) in forward and backward directions. Translation of the surface resulted in displacement of the COG, in response to which participants were instructed to restore their balance [18]. Each size of platform translation in forward and backward direction was offered 3 times, randomly ordered with a random time interval. Latency times (ms) and response strength (°/s) were measured for each of the 3 trials and averaged for the 6 combinations of the size and direction of platform translation. The response strength reported the participants' active response at each size and direction of the translation, defined as the amount of angular momentum needed to counteract sway (approximately twice the angular momentum of the platform in opposite direction) induced by the platform translation. Low response strength represented adequate amplitude scaling in response to platform translations [18].

The Rhythmic Weight Shift (RWS) test evaluated the voluntary ability to move the COG from right to left and forward to backward between two targets (preset at 50% at the measured LOS of the participant) at slow (3 seconds peak to peak), medium (2 seconds peak to peak), and fast (1 second peak to peak) pacing [11]. Movement velocities of the target were 2.67°/s (slow mediolateral), 4°/s (medium mediolateral), 8°/s (fast mediolateral), 1.78°/s (slow anterior-posterior), 2.68°/s (medium anterior-posterior), and 5.35°/s (fast anterior-posterior). Participants were instructed to move the cursor towards a star on the screen by moving their pelvis (COG) left/right or forward/backward without moving their feet or other body parts. The outcome variable was directional control. This is a ratio of the amount of movement in the intended direction to the amount of deviation from the ideal movement trajectory. It is expressed as a percentage, calculated for every combination of movement direction and speed. The percentage reflects the average score of 6 movement repetitions in one plane (as shown in Figure 4). Higher scores indicate better directional control.

### 2.3. Statistical Analysis

Overall group differences in the freezer, nonfreezer, and control group for descriptive variables (gender, age, and length), SOT (equilibrium score and postural strategy), MCT (latency time and response strength), and RWS (directional control) outcome measures were analyzed using the nonparametric Kruskal-Wallis (K-W) ANOVA by ranks test due to small sample size and abnormal distribution of data. Post hoc Mann Whitney *U*-tests (M-WU) were used to compare individual between-group differences for clinical variables (between patient groups), SOT, MCT, and RWS. Additional within-group analyses for the MCT were carried out using the Wilcoxon matched pairs test. All tests were performed using Statistica (Statistical analysis Software, version 8) at an *α*-level of 0.05.

## 3. Results

Demographic characteristics of both groups are shown in Table 1. A significant difference was found between freezers and nonfreezers on FOGQ scores (*P* = 0.006). Freezers showed a higher frequency of falls (4/9) and near falls (5/9) in the preceding 3 months compared to nonfreezers (1/10 and 3/10, resp.) (*P* = 0.008). No significant differences were found for UPDRS score (part III, motor subscale), MMSE score, and disease duration between subgroups. Nevertheless, freezers had a twofold longer median disease duration compared to nonfreezers. Knee proprioception mean scores were significantly worse in freezers (2.4°) compared to controls (1.6°) (*P* = 0.02), but not to nonfreezers (*P* > 0.05).

Patients were taking their normal daily doses of anti-Parkinson medication. Eight of 9 freezers took levodopa with a mean active dose of 359.72 mg/day. Nine out of 10 freezers took levodopa with a mean active dose of 315 mg/day. Other medication intake was not significantly different between groups.

### 3.1. Automatic Postural Control: SOT-Test

Equilibrium scores showed a significant difference between the 3 groups for SOT6 (K-W: *P* = 0.008). Post hoc analysis indicated that both freezers and nonfreezers had lower equilibrium scores for SOT6 compared to controls (M-WU: *P* = 0.02;  *P* = 0.005), but no PD subgroup difference was found. No significant overall group differences were found for SOT1–SOT5 (Table 2). All groups showed very similar and minimal sway in all conditions, even during conditions where the balance platform was moving and sensory information was compromised (SOT4–SOT6). In SOT5 and SOT6, data were omitted if a near fall occurred which was prevented by the tester. Two freezers and 1 nonfreezer tended to fall during each trial of SOT5 and 1 control did so during 1 trial of SOT5. In addition, 3 freezers almost fell during 1 or more trials of SOT6, similar to 3 nonfreezers and 5 controls.

**Figure 1:**
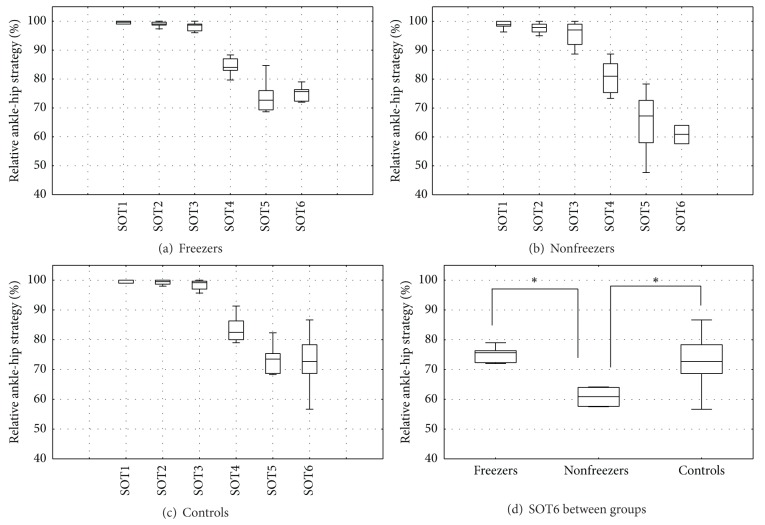
Group comparison of ankle-hip strategy in SOT conditions. Boxes represent median values and interquartile ranges (Q25–Q75), with error bars indicating the nonoutlier ranges. Panels (a), (b), and (c) show the relative ankle-hip strategy (with 100% ankle strategy only and 0% hip strategy only) for each SOT test. Panel (d) shows the relative ankle-hip strategy for SOT6 between freezers, nonfreezers, and controls. *Significant difference (*P* < 0.05) between two groups (post hoc).

The postural control strategy used by participants to maintain balance during the SOT is shown in Figure 1. An overall group comparison showed a significant difference between groups for SOT6 (K-W: *P* = 0.02). Freezers (75.67% ± 4) and controls (72.67% ± 9.67) relied significantly more on the ankle strategy compared to nonfreezers (60.92% ± 6.33) in SOT6 (M-WU: freezers: *P* = 0.02; controls: *P* = 0.02). There were no significant differences between groups for SOT1, SOT2, SOT3, SOT4, and SOT5. In general, all groups increased the amount of hip strategy from SOT1 to SOT6.

### 3.2. Automatic Postural Control: MCT-Test

**Figure 2:**
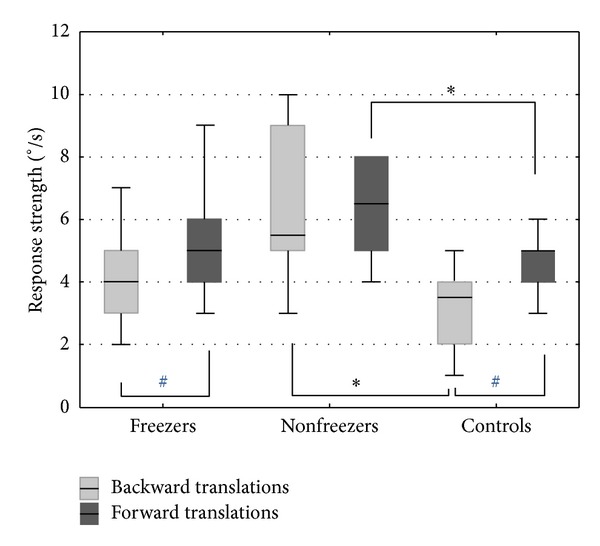
Group differences in MCT performance. Response strength of the left leg in forward and backward translations during MCT (pooled for small, medium, and large conditions). Boxes represent median values and interquartile ranges (Q25–Q75) with error bars indicating the nonoutlier range. *Significant difference (*P* < 0.05) between two groups (post hoc), ^#^significant difference (*P* < 0.05) within groups.

Figure 2 displays the response strength to translations during the MCT. As the results for both legs were very similar, the data are presented for one leg only.

**Figure 3:**
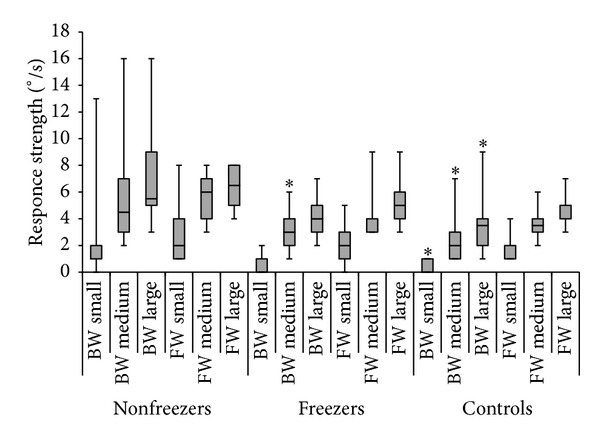
Group differences in MCT performance per condition. Response strength of the left leg in forward (FW) and backward (BW) translations for small, medium, and large conditions separately. Boxes represent median values and interquartile ranges (Q25–Q75) with error bars indicating range. *Significant difference (*P* < 0.05) with nonfreezers (post hoc).

Statistical testing indicated overall group differences in response strength for backward translations in the 3 conditions (K-W: small: *P* = 0.02; medium: *P* = 0.01; large: *P* = 0.03). Between-group analysis showed that, in all backward conditions, the response strength of the nonfreezer group was larger than that of the freezer and control groups, indicative of poorer automatic postural control in nonfreezers (Figure 2). Backward translations brought on significantly stronger responses in nonfreezers than in controls during small (M-WU: *P* = 0.007), medium (M-WU: *P* = 0.005), and large (M-WU: *P* = 0.01) translations (Figure 3). Comparing freezers and nonfreezers, a significant difference was found in the medium backward translation (M-WU: *P* = 0.047) in which nonfreezers showed larger responses. No significant differences were found between freezers and controls.

### 3.3. Voluntary Postural Control: RWS-Test

**Figure 4:**
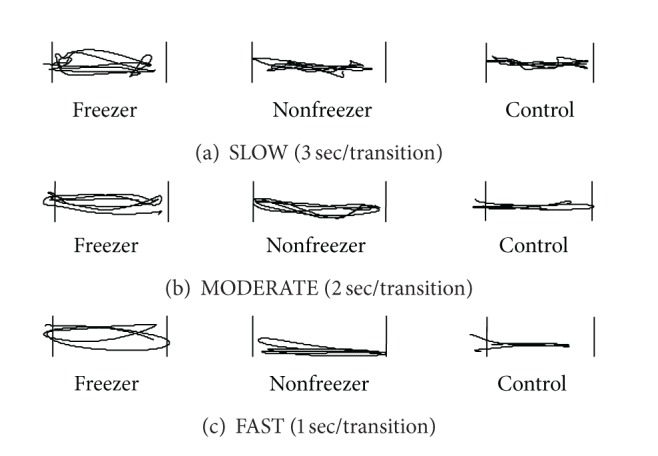
COG pattern during mediolateral RWS. Each graph shows a representative COG movement pattern for a freezer, nonfreezer, and control participants. The vertical bars indicate the distance of the shift the participants were instructed to make and are set at 50% of the participants' limit of stability. Panels (a), (b), and (c) show the COG pattern when a cue was given with respectively a 3 second, 2 second, and 1 second interval.

The RWS test was utilized to gain insight in the voluntary intentional shifting of the COG. Figure 4 shows an example of the movement pathway in the mediolateral direction of a representative participant of each group. It shows that freezers performed worse and had a more irregular pathway compared to nonfreezers and controls.

Statistical analysis revealed overall group differences for directional control in the moderate mediolateral direction (K-W: *P* = 0.01) and the slow, moderate, and average anterior-posterior direction (K-W: *P* = 0.002, *P* = 0.05, *P* = 0.006).  Table 3 shows the results of the between-group analysis indicating significantly less directional control for the slow anterior-posterior shift in freezers compared to nonfreezers (M-WU: *P* = 0.02) and controls (M-WU: *P* = 0.0009). Additionally, freezers showed significantly less directional control for average anterior-posterior (M-WU: *P* = 0.002) and average mediolateral (M-WU: *P* = 0.02) shifts compared to controls. There were no significant differences between nonfreezers and freezers except for the slow anterior-posterior condition in which nonfreezers had better directional control. No significant differences were found in movement velocity between groups.

## 4. Discussion

The purpose of this study was to investigate for the first time differences in voluntary and automatic postural control between freezers, nonfreezers, and controls to gain insight in the connection between postural control deficits and freezing of gait (FOG).

In contrast to our hypothesis, we found that freezers did not have a greater problem with automatic postural control compared to nonfreezers, even in situations with both unreliable visual and proprioceptive input. This points to a similar ability to integrate sensory information during quiet stance in patients with freezing compared to patient without freezing. Contrary to the lack of differences in response strengths between freezers and nonfreezers, we did find a significant difference between PD subgroups for the strategy used to maintain balance. A shift from an ankle to hip strategy is normal when changing from quiet stance in stable conditions to situations where balance becomes compromised [19]. Freezers showed similar strategies to maintain balance compared to controls throughout the SOT. Nonfreezers showed less ankle strategy during SOT6 compared to freezers and controls, which indicates more balance problems in both normal balance condition and condition where vision and proprioception (SOT6) are compromised. These results are surprising and suggest that, in this sample, nonfreezers had poorer postural control or more difficulty with sensory integration to maintain postural control compared to freezers and controls. However, because of the lack of differences in other SOT conditions, this conclusion cannot be generalized. Contrary to our results, Tan et al. [20] reported that freezers show a greater proprioceptive deficit compared to nonfreezers and controls in a force target task with a tendon vibration protocol. When we assessed proprioception separately by the lower limb position sense test, we found larger errors for freezers compared to controls, but not compared to nonfreezers. Combined with the SOT results, this favours the conclusion of poorer postural control in nonfreezers compared to freezers and controls. However, we did not separately assess other sensory modalities.

Nevertheless, similar results were found during the MCT. There were no significant differences in response strengths between freezers and nonfreezers except for the backward translation of medium size, confirming that nonfreezers tended to have less adequate postural control. In addition, there were significant differences in response strengths between nonfreezers and controls in almost all forward and backward translations. The more normal pattern of response strengths in freezers may be explained by an increased alertness of freezers to the possibility of losing balance. Snijders et al. [21] showed that freezers anticipated an upcoming obstacle more quickly during treadmill gait. Nonfreezers on the other hand may have expected the perturbations less, leading to exaggerated response strengths. We found no response time differences between freezers and nonfreezers during the MCT, indicating no movement initiation differences between groups.

Overall, the pattern that nonfreezers had more impaired postural control is particularly notable, given that UPDRS and H&Y scores were similar between groups and that freezers tended to have longer disease duration. Freezers were taking a higher levodopa dose (not significant), which is consistent with the contention that FOG may be relatively less levodopa responsive than other PD symptoms [22]. The fact that the present exploratory study was conducted in “on” phase may explain these findings since a higher levodopa dose may have contributed to the better balance in freezers compared to nonfreezers. However, other studies [23, 24] showed no significant improvement in postural control with levodopa treatment and even increased postural sway in levodopa-treated patients.

The RWS task was used to test the participants' voluntary ability to move the COG in an intended direction at different velocities [11]. In this task, freezers had strikingly worse directional control compared to the other groups and more so in the anterior-posterior than in the mediolateral direction. Nevertheless, they were able to perform the weight shifts at an adequate speed, and therefore this deficit cannot be interpreted as an expression of bradykinesia. When performing the intended movement trajectories, freezers may have opted to prioritize optimal velocity resulting in neglect of adequate directional control [25]. Another study suggested that patients with PD display a speed/accuracy tradeoff during repetitive movement tasks, but this has never been shown to be more present in freezers [26]. In addition, the task involved a visual target, which may have served as an external cue. Freezers and nonfreezers are known to increase gait speed in response to an external trigger. The impaired voluntary COG control, particularly in the sagittal plane, may be a contributing factor to loss of balance during freezing and festination when patients cannot counteract the forward propulsion inherent to hastening of gait. In this respect, Bloem et al. [1] suggested that this pattern, followed by a sudden arrest of walking, may be one of the reasons why falling and freezing are related [1].

Interestingly, in controlled situations (SOT and MCT), no differences were found in “fall frequency” (representing the number of uncompleted trials) between freezers, nonfreezers, and controls. This may point to the fundamental deficit of automatic motor control in PD, which is difficult to capture during laboratory testing. The fall frequency questionnaire showed that 4/9 of the patients in the freezer group fell during the past three months in daily life compared to 1/10 of the nonfreezers, which is in line with earlier work [1, 3, 8].

Several limitations of the study should be taken into account when interpreting the present findings. The relatively modest differences in balance performance between freezers and nonfreezers found in our exploratory study may be related to the small sample size and could have underestimated actual differences between freezers and nonfreezers. In addition, no multiple testing corrections were applied because of the hypothesis-generating nature of this study. Furthermore, all tests were done in the “on” state, providing insufficient contrast between both patient groups. However, several studies have reported that patients still experience a deterioration of balance when they are in the “on” state [24, 27]. We only studied limited aspects of postural control, not taking into account other components like balance during gait tasks, lower extremity strength, and ankle range of motion. Therefore, to fully elucidate the differences in postural control between freezers and nonfreezers we recommend that future studies be conducted in larger sample sizes in both “on” and “off” state. Additionally, we suggest using appropriate multiple testing corrections and including different aspects of postural control to fully understand postural control problems in patients with PD with and without freezing. Finally, the significant difference in gender distribution between groups could have influenced our results as previous research has shown differences in postural sway between men and women [28].

## 5. Conclusion

Freezers performed better than nonfreezers on a balance platform requiring sensory integration and response to unexpected translations. They did show a particular impairment in voluntary weight shifting, mainly in the anterior-posterior direction. Future research is needed to pinpoint differences in automatic postural control between freezers and nonfreezers and to unravel whether proprioceptive deficits underlie these problems. Additionally, it needs to be elucidated why patients with freezing have a higher fall frequency.

## Figures and Tables

**Table 1:** Clinical variables of nonfreezers, freezers, and controls.

	Nonfreezers (*n* = 10)	Freezers (*n* = 9)	Controls (*n* = 10)	*P* value
Gender (m/f)	10/0	7/2	3/7	0.003*
Age (y)	68 (58–75)	65 (62–73)	66 (63–74)	0.90
Height (cm)	174 (169–178)	173 (166–176)	174 (168–179)	0.90
H&Y				0.49
H&Y 2	4	1	NA	
H&Y 2.5	2	5	NA	
H&Y 3	4	2	NA	
H&Y 4	0	1	NA	
DD (y)	6 (5–8)	12 (10–14)	NA	0.09
FOGQ tot (0–28)	2.5 (2–4)	13 (6–14)	NA	0.006**
MMSE (24–30)	29 (28–30)	29 (28–30)	NA	0.83
Fall frequency	1/10	4/9	NA	0.008**
UPDRS (III) (0–108)	25.5 (19–27)	26 (23–28)	NA	0.71
Knee proprioception^#^	2.4 (1.8–3.6)	2.2 (2-3)	1.6 (1-2)	0.02*

Median and 25th percentile and 75th percentile (Q25 and Q75) are presented between brackets.

^#^Larger difference in degrees indicates greater difference between right and left leg and thus greater proprioceptive deficit.

Abbreviations: H&Y: Hoehn and Yahr stadium; DD: disease duration; FOGQ tot: total score of the freezing of gait questionnaire; MMSE: mini mental state examination; UPDRS: unified Parkinson's disease rating scale; NA: not applicable; *significant difference (K-W: *P* < 0.05) between 3 groups (group effect); **significant difference (M-WU: *P* < 0.05) between freezers and nonfreezers (post hoc effects).

**Table 2:** SOT equilibrium score descriptive variables.

Test	Freezers	Nonfreezers	Controls	*P* value
SOT1	94.3 (92.7–94.7)	93.2 (92.0–94.3)	94.3 (93.0–96.0)	0.20
SOT2	92.0 (90.7–92.3)	89.3 (88.0–93.0)	92.2 (90.0–93.7)	0.52
SOT3	90.0 (87.7–92.3)	88.0 (88.0–92.0)	89.7 (88.3–91.7)	0.82
SOT4	75.7 (70.7–84.3)	74.8 (68.3–82.0)	75.5 (65.7–81.3)	0.82
SOT5	55.0 (43.0–69.3)	55.0 (48.3–61.3)	56.2 (50.7–69.0)	0.61
SOT6	54.0 (52.0–65.0)	53.0 (42.3–58.0)	58.9 (58.0–61.0)	<0.01*

Estimated median interquartile range of the SOT equilibrium scores is presented in percentage (%). ∗Significant differences (*P* < 0.05) for Kruskal-Wallis ANOVA overall group analysis.

**Table 3:** Directional control during rhythmic weight shift.

	Freezers	Nonfreezers	Controls	*P* value (Freezers versus nonfreezers)	*P* value (Freezers versus controls)	*P* value (Nonfreezers versus controls)
Slow mediolateral	77 (62–77)	74.5 (69–81)	79.5 (76–83)	0.24	0.07	0.28
Moderate mediolateral	74 (71–79)	83 (76–86)	86.5 (82–88)	0.07	<0.01**	0.35
Fast mediolateral	84 (77–87)	88.5 (87–90)	87 (83–91)	0.13	0.24	0.80
Average mediolateral	79 (71–82)	80.5 (78–96)	83.5 (81–85)	0.13	0.01*	0.48
Slow anterioposterior	49 (22–67)	70 (62–79)	77 (72–83)	0.02*	<0.01**	0.089
Moderate anterioposterior	61 (38–77)	75 (70–80)	82.5 (66–87)	0.16	0.01*	0.25
Fast anterioposterior	76 (64–82)	81 (68–82)	82.5 (79–87)	0.50	0.07	0.39
Average anterioposterior	62 (43–74)	73 (70–79)	81 (76–84)	0.09	<0.01**	0.052

Estimated median interquartile range of the directional control is presented in percentage (%). *Significant difference (*P* < 0.05); **significant difference (*P* < 0.01). Measurement unit is % of optimal performance.
